# Supplementary material for: Phylodynamics of SARS-CoV-2 Lineages B.1.1.7, B.1.1.529 and B.1.617.2 in Nigeria Suggests Divergent Evolutionary Trajectories
Source: Pathogens. 2025 Oct 26;14(11):1091. doi: 10.3390/pathogens14111091 (PMC12655259; doi:10.3390/pathogens14111091)
Supplement: Supplementary file 1 [file pathogens-14-01091-s001.zip › pathogens-3898174-supplementary.pdf]

SUPPLEMENTAL TABLE

Data Availability

GISAID Identifier: EPI_SET_250911ba
DOI: https://doi.org/10.55876/gis8.250911ba

All genome sequences and associated metadata in this dataset are published in GISAID's EpiCoV database. To view the contributors of each individual sequence with details such as accession number, Virus name, Collection date, Originating Lab and Submitting Lab and the list of Authors, visit EPI_SET_250911ba

Data Snapshot

EPI_SET_250911ba is composed of 1,903 individual genome sequences.
The collection dates range from 2020-08-03 to 2022-07-16;
Data were collected in 1 countries and territories.
